# A Deep Learning Based Data Recovery Approach for Missing and Erroneous Data of IoT Nodes

**DOI:** 10.3390/s23010170

**Published:** 2022-12-24

**Authors:** Perigisetty Vedavalli, Deepak Ch

**Affiliations:** School of Electronics Engineering, VIT-AP University, Inavolu, Beside AP Secretariat, Amaravati 522237, India

**Keywords:** IoT node, spatial-temporal correlation, data reliability, hierarchical LSTM, data recovery, missing data, and erroneous data

## Abstract

Internet of things (IoT) nodes are deployed in large-scale automated monitoring applications to capture the massive amount of data from various locations in a time-series manner. The captured data are affected due to several factors such as device malfunctioning, unstable communication, environmental factors, synchronization problem, and unreliable nodes, which results in data inconsistency. Data recovery approaches are one of the best solutions to reduce data inconsistency. This research provides a missing data recovery approach based on spatial-temporal (ST) correlation between the IoT nodes in the network. The proposed approach has a clustering phase (CL) and a data recovery (DR) phase. In the CL phase, the nodes can be clustered based on their spatial and temporal relationship, and common neighbors are extracted. In the DR phase, missing data can be recovered with the help of neighbor nodes using the ST-hierarchical long short-term memory (ST-HLSTM) algorithm. The proposed algorithm has been verified on real-world IoT-based hydraulic test rig data sets which are gathered from things speak real-time cloud platform. The algorithm shows approximately 98.5% reliability as compared with the other existing algorithms due to its spatial-temporal features based on deep neural network architecture.

## 1. Introduction

IoT has a wide range of applications, and the data which are gathered from various IoT nodes contain missing values due to unreliable links and unexpected damages [1]. Missing data lead to inaccuracy and unreliability, which influence the behavior of IoT applications. Analyzing and collecting data from IoT nodes cannot only consider the state of the network, but it can also help to make appropriate decisions during catastrophic situations. Therefore, data reconstruction methods should be required for maintaining network reliability and fault tolerance [2,3]. An IoT-based nuclear monitoring application requires the collection of data continuously a small data error may lead to serious consequences. This is the best example to show the prominence of data recovery approaches in IoT applications. Data recovery approaches can help to avoid catastrophic conditions if any difficulties occur. Table 1 gives the nomenclature which is used in the following sections.

Data re-transmission is one of the familiar approaches to reducing data missing. In this approach, if any data are missing from the particular node, data can be transmitted until it transmits successfully. Mainly, there are two limitations when using this approach in IoT networks. IoT nodes are low-power devices, re-transmission of data requires additional power usage and data re-transmission leads to latency due to traffic between transmitter and receiver ends. Therefore, in this case, data recovery approaches and sparse sampling [4] is one of the energy-efficient methods to eliminate the data missing problem in sensor networks. The data aggregation method will be used to eliminate the redundant transmission of data. The perceptually important points-based data aggregation method [5], a data gathering and aggregation technique with selective transmission [6], is utilized to avoid redundant transmission of data which leads to enhance the 93% and 94.6% of energy consumption and accuracy for the application. In [7], a minimum description and compression technology have been utilized for the real-time transmission of data of sensors to build up the remote monitoring of the IoT-based agriculture system. However, this technique reduces the transmission speed of data. Data recovery is the reconstruction or recollection of data based on previously recorded data points. Statistics (median, mean) is one of the easy methods to extract missing data with the help of existing data [8]. However, interpolation or statistical methods cannot predict and recover the data accurately. The data recovery approach has the ability to analyze the node sensing data structure and evaluate a few main factors which are helpful for the data recovery process are mentioned as follows:

- Time correlation—data have dependency and periodicity on their historical node data.
- Spatial Correlation—in the network, every node has a dependency on its neighbor node data.
- Data quality control—the node data are lost due to noise and compared with original values for maintaining the data quality with the help of data pre-processing.

In dynamic recurrent neural networks, data are associated with time and the next output is produced after a time step. Time is the essential component of these models. Recurrent networks can allow feedback loops including self-feedback loops. Data can be propagated through forward and backward feedback between the nodes and self-feedback for the node is also allowed. Deep learning approaches can able to predict the missing data accurately, so these approaches have been used frequently for data reconstruction in recent years [9]. Among all the algorithms hierarchical long short-term memory (HLSTM) is one of the most powerful deep learning algorithms. It has deep architecture and also having powerful self-learning capability features. HLSTM is stacked with many LSTM layers which helps for accurate prediction hierarchically.

LSTM emulates human beings’ memory process, which means it remembers the signal step by step and grabs every cell’s data. In LSTM, the neural network has input, output, forget, and memory gates for processing data. The gates continuously trace the quantity of data that is transmitted from the input to output gates and also from one cell to another. Initially, the input gate regulates the value of the input, that is carried to the next cell. The forget gate memorizes only necessary data and eliminates all the unwanted data, and then transmitted it to the following cell. The memory gate needs to store all the required information and the output gate determines the information from the input and that information is used as output for previous gates, it results that LSTM neural network cell serves as its memory, assisting in the extraction of long-term dependencies in the input but it cannot be able to do feature extraction properly. LSTM is a recurrent neural network and has better prediction and processing of long-term time series events [10,11,12]. LSTM has been used in a wide range of applications for various types of data prediction. In [13], empirical mode decomposition with LSTM has been utilized for stock prediction and also check-up with closing global stocks data. In [14], the authors proposed a best deep learning architecture with a dual-step feature selection algorithm for the prediction of indoor CO_2_ concentrations for various spaces in a building. In [15], a weather conditions prediction model is implemented with the help of transductive learning along with LSTM.

HLSTM is stacked with two LSTM layers in the proposed method for better prediction and feature extraction. The main concept of HLSTM consists of the nonlinear mapping of inputs and outputs layers and these layers are used for hierarchical feature learning. The model can understand the properties of raw ST signal from various aspects at every time step by stacking multiple LSTM layers. Among all the algorithms, hierarchical long short-term memory (HLSTM) is one of the most powerful deep learning algorithms. It has deep architecture and also has powerful self-learning capability features. HLSTM is stacked with many LSTM layers which helps for accurate prediction hierarchically.

In this paper, a novel clustering algorithm with ST-HLSTM has been proposed for missing and erroneous data recovery. The proposed approach is simulated at the cluster head due to the constraints of IoT nodes. To recover the missing and erroneous data, initially, a clustering algorithm is proposed to cluster the nodes based on their ST correlation. The data which are gathered from the IoT nodes at each cluster is generated as a raw matrix. Each row in the raw matrix is represented as temporally correlated readings and the column consists of spatially correlated data of IoT nodes. The raw matrix is the input for HLSTM to recover the missing data. The contributions towards this research are summarized as follows

- The proposed method is applied to both static and dynamic IoT networks. A clustering algorithm is used to divide the IoT nodes into various clusters, based on their ST correlation.
- The proposed approach has been simulated in real-world sensory data sets, which resulted in more than 3% of reliability as compared with other data recovery approaches. Those are bidirectional long short-term memory (BI-LSTM) [16], matrix factorization alternating least squares (MF-EALS) [17], and data reconstruction using temporal stability guided matrix completion (DRTSMC) [18] recovery approaches. The existing approaches utilized redundancy and periodicity to predict the specific node data depending on the past data, resulting in low prediction stability and biased predictions. Due to its long-term dependencies on time series data depending on long-time interval events, the proposed approach uses HLSTM because of its strong prediction stability. Data correlation between the node data will be useful to recover erroneous and missing data. By fusing HLSTM with Spatial and temporal correlation the prediction model improves data reliability as compared with existing approaches.

### Motivation behind the Work

In some IoT applications such as IoT-based gas, nuclear reactors, weather, and earthquake monitoring systems the IoT nodes need to be deployed in remote and harsh environments. Generally, IoT nodes are having energy constraints because nodes are working with limited energy devices, so maintenance of such types of applications is very hard. Due to environmental factors and depletion of energy, the node may fail automatically. In this scenario, the node sensing data may be lost. The user cannot able to access the data for a long time. Therefore, the important information needs to access or collected before the node failure. This problem can be resolved with the help of data recovery approaches in IoT applications.

The rest of the paper is organized as follows, Section 2 presents a brief review of existing data recovery approaches. Section 3 demonstrates the proposed approach comprehensively. The simulation of existing approaches and comparative results are discussed in Section 4. Section 5 discussed the conclusion and future scope of this work.

## 2. Related Work

Data integrity is the most essential component, which influences the overall IoT network performance. IoT is utilized in many critical and safety applications, such as hospital safety management systems, nuclear stations, smart transport systems, industries management, etc. Recently, IoT has also been employed for monitoring the performance of high-speed heterogeneous networks [19]. In [20] an extreme machine learning approach is proposed to predict the missing data for micro-climate detection applications. This approach is more accurate in prediction with high speed for only short time series data.

The spatial approach is utilized to extract the relationship between the spatially correlated node data at a similar sensing interval. The temporal approach leverages the temporal relation between the same node recorded data. The most common interpolation method, k-nearest neighbor, along with the linear regression model, is used for missing data reconstruction in [21,22].

DRTSMC approach is proposed in [18] which has been utilized for data reconstruction and it is initiated with the formulation of a matrix based on the temporal stability between the nodes in the network. To reduce the errors during the data reconstruction a splitting technique and a block coordinate descent method are developed accordingly. Based on the spatial correlation an auto-regressive model has been developed to recover the missing data [23]. The auto-regressive coefficient is continuously updated until the model meets the required missing data imputations. A missing data imputation with a continuous iteration algorithm is proposed in [24]. Missing data imputation is a process of segmenting a large time-series gap between the data into multiple pieces. To acquire reliable data reconstruction, the iteration procedure needs to run continuously. This algorithm is applicable only when a large amount of data is missing. In [25], authors proposed a missing data imputation algorithm based on the random forest which is called a miss forest. This algorithm can able to predict the accurate missing values with the help of statistical models. In [26], the authors have collected data from multiple heterogeneous outdoor weather monitoring sensors. The data set has been utilized for supporting and bench-marking various data-driven approaches to impute the missing sensor data. In [27], the authors proposed an advanced deep learning algorithm based on generative adversarial neural networks for missing data imputation. In [28], the authors proposed an algorithm to predict the incomplete fields in the vehicle parking duration in the survey data. The authors implemented a data recovery approach for a fault-tolerant IoT node (DRAFT) algorithm based on a redundant array of independent disks (RAID5) structures in [29] to recover the missing data for a fault-tolerant IoT node. Missing data can be recovered with the help of direct neighbor nodes in the cluster. This algorithm can able to recover the single node data at an instance algorithm is utilized for data recovery efficiently. Sparsity constraints were extracted through the temporal feature of data and a dual-stage matrix completion algorithm is proposed in [30] for missing and corrupted data recovery.

A partial canonical identity missing data recovery (PCIMDR) algorithm with discrete cosine transform (DCT) has been implemented in [31,32]. Initially, data are encapsulated into a matrix then the missing data can be identified through PCI and the DCT is used for data recovery. To recover the missing time series data MF-EALS [17], matrix minimization, ST correlation with low-rank matrices [33], and matrix completion [34] methods are implemented in sensor networks. Depending on the data patterns and correlation among the time series data the missing data have been recovered. To reduce the complexity of the matrix a least square algorithm is utilized. The least-square algorithm results in high accuracy, but it is highly difficult to implement in complex networks. The authors proposed a spatial-temporal correlation framework that depends on convolution neural networks [35], in this paper, the authors mainly concentrated on three types of missing data in remote sensing networks eliminating the deadlines from the Aqua MODIS 6th band, scan lines in the land set enhanced thick clouds and Thematic Mapper Plus and data can be recovered for sensing networks.

The Bi-LSTM, model is utilized to recover the missing data in sequence to sequence deep learning-based wireless sensor network (WSN) architectures [16]. This model will help to recover the missing data based on both future and past information. The authors proposed a deep neural network helps to detect unpredictable and unseen attacks in the network [36]. The authors proposed convolution neural networks (CNN) in [37,38] for missing data detection and reconstruction. Initially, a non-linear relationship is drawn between the missing values and those are trained with all incomplete and complete signals. Then CNN algorithm will initiate the recovery process. In [39], the authors implemented a novel approach by combining the support vector machine (SVM), Refining support vector machine (RSVM), and optimizing support vector machine (OSVM), for recovering the missing data in IoT networks. Table 2 shows the comparison of existing data recovery approaches with the proposed approach.

The authors proposed a reordering algorithm combined with compressive sensing [40,41,42] for the reconstruction of missing data. This method improves the sparsity of the signals by compressing the measurements required for the data reconstruction. In [43,44], a dual prediction algorithm with adaptive sensing is utilized in distributed networks for data recovery. The data have been reduced at the node level with the help of adaptive sensing and reconstruction of data can be conducted with a dual prediction algorithm with high energy efficiency. Tensor-based methods such as high accuracy low-rank tensor completion algorithm (HaLRTC) [45,46], Vector Auto-regressive Model Based Imputation (VAR-IM) model with vector auto-regressive model algorithm and alternating direct method of matrix data reconstruction (ADMMR) [47] are used to reconstruct the multi-attribute and multi-variate time series sensor data. These methods cannot able to predict the data accurately when the missing data rate is more than 60%. In [48], based on sensory data features such as multi-attribute correlation, low rank, space similarity, and time stability is analyzed through environmental space-time improved compressive sensing technique, and missing data are estimated through the multi-attribute assistant algorithm.

**Table 2:** Comparison of various data recovery approaches with the proposed approach.

Author	Method	Single/Multi Attribute	Data Recovery Prediction	Complexity
L. Chen [18]	Iterative multiple segmented gap	Single attribute	Similar nodes data	High
Y. Liu [24]	Joint sparsity Multi-attribute and low rank Constraints	Multi-attribute	Similar nodes data	Low
Vedavalli [29]	RAID Structures	Single attribute	Similar nodes data	High
X. Therefore, ng [17]	Matrix Factorization	Single attribute	Similar nodes data	High
X. Yu [33]	Sequence to sequence Imputation method	Single attribute	Similar nodes data	High
Y.-F. Zhang [37]	BI-LSTM	Single attribute	Similar nodes data	High
G. Chen [42]	Compressive Sensing	Single attribute	Multiple nodes data	High
Proposed approach	ST-HLSTM	Multi attribute	Multiple nodes data	High

### Theory of HLSTM

Deep neural architectures have demonstrated their strong potential in feature self-learning. Recurrent neural networks (RNN) were initialized to sort out the time series problems. These are completely different from traditional neural networks which are set up by multiple-layer perception which can depict the input data to focused vectors. RNN can able to trace back the complete history of previous inputs. The traditional RNNs cannot capture the long-term dependencies to overcome this problem LSTM was introduced. The basic idea of the LSTM depends on the multiple gates which are utilized to control the flow of information in a time series manner with good accuracy. At each time step, ‘*T*’ hidden state ‘$h_{T}$’ is updated with fusion data at the same time step ‘$x_{T}$’, output gate ‘$o_{T}$’, forget gate ‘$f_{T}$’, input gate ‘$i_{T}$’, memory cell ‘$c_{T}$’ and last time step hidden state ‘$h_{T-1}$’.

**Figure 1:**
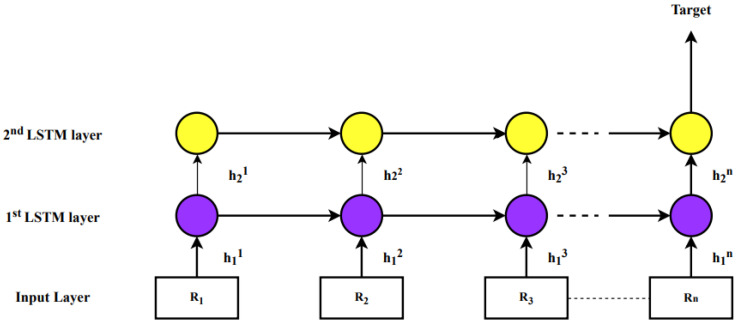
Structure of Hierarchical LSTM.

HLSTM is the flavor of LSTM, it is stacked with multiple LSTM layers, and also feature learning has been conducted hierarchically. The output of the first LSTM hidden layer is not only the input of the next hidden layer of LSTM and also propagated forward through time as shown in Figure 1. The final equations for each gate are given as follows

(1) $$i_{2}^{t}=\sigma \left(w_{2}^{i}h^{t}+v_{2}^{i}h_{2}^{t-1}+b_{2}^{f}\right)$$

(2) $$f_{2}^{t}=\sigma \left(w_{2}^{f}h^{t}+v_{2}^{f}h_{2}^{t-1}+b_{2}^{f}\right)$$

(3) $$o_{2}^{t}=\sigma \left(w_{2}^{o}h^{t}+v_{2}^{o}h_{2}^{t-1}+b_{2}^{o}\right)$$

(4) $$c_{2}^{t}=f_{2}^{t}⊙c_{2}^{t-1}+i_{2}^{t}⊙\tanh\left(w_{2}^{c}h^{t}+v_{2}^{c}h_{2}^{t-1}+b_{2}^{c}\right)$$

(5) $$h_{2}^{t}=o_{2}^{t}⊙\tanh\left(c_{2}^{t}\right)$$

For the first layer, the input is raw data, and the output of the first layer is the extraction of raw signals, which are considered hierarchical features. Other layers are utilized the previous layer’s output as input, and the second LSTM layer output is used as classification. By stacking multiple LSTM layers there is an advantage and a few of them are listed as follows:

- Stacked LSTM layers empower the proposed model to learn various characteristics of raw data in different perspectives at a unit time step.
- Proposed model parameters are allocated over the complete space of the model without enhancing memory space which empowers the model to improve non-linearity and convergence of raw data.
- Proposed model is stacked with two LSTM layers which enable hierarchical feature learning. Note that the HLSTM network can handle memory with time steps. The proposed model which understands the pattern of raw data memorizes it, catches the important points, and based upon that will predict the missing data.

## 3. Methodology

The network has been trained using the gradient descent error optimization method. The error is propagated back to all previous time steps to optimize the weights. Hence the process is called back propagation through time. Once the network is trained, it can predict the next sequence based on the previous sequence data as inputs.

### 3.1. Data Pre-Processing

The paper utilizes hydraulic-test rig data set which has been collected from 51 nodes that are used to study missing and erroneous data prediction problems in the IoT. The data sets hold around 3.5 million sensory node data gathered from 51 nodes including date, timestamp, temperature, pressure, and volume flows of the valve, accumulator, pump, and cooler hydraulic machine which are continuously varied.

In sensor networks, missing and erroneous data are due to two main reasons one is errors occur during data transmission and node failure. To avoid the impact of abnormal data on the erroneous and missing data prediction, here dealing with two types of data outliers: local and global outliers for quality prediction. The data samples—which are in the range of the data set, but deviated from the neighbor node’s data samples—are considered local outliers. The samples which are completely deviated from the other data samples in the data set are considered global outliers.

### 3.2. Local and Global Outliers Processing

These two outliers have a great impact on feature extraction and data normalization. Therefore, these outliers should be removed with the help of the quartile method in this paper. Initially, need to detect the upper quartile *q*_1_, median *q*_2_, and lower quartile *q*_3_ from the data set. Then evaluate the inter-quartile range by subtracting the lower quartile from the upper quartile (IQR = *q*_1_ − *q*_3_). Lastly calculating the lower fence and upper fence which are considered as upper and lower bounds of the data set. The data samples that are falling out of the range are treated as a global outlier and the adjacent neighbor node collecting the same attribute data samples is considering them as a local outlier.

### 3.3. Data Correlation Analysis

The hydraulic test rig data set consists of various sensor data gathered by 51 nodes. To choose appropriate sensory data for training the neural network. To make accurate missing and erroneous data predictions common neighbor nodes need to be extracted through the clustering phase. Min–max data normalization technique is utilized for linear transformation of data in between [0, 1] and given as follows Equation (6)

(6) $$d^{\prime }=\left(d-d_{min}\right)/\left(d_{max}-d_{min}\right)$$

where *d* is the original data samples, $d_{min}$ is the minimum of the data samples, $d_{max}$ is the maximum of the data samples, and finally *d*’ is the normalized data sample. The data can be mapped to [0, 1] after the normalization process. The Spearman correlation coefficient method is utilized to quantify the correlation among the various nodes in the network which are considered as CNN of the other nodes.

**Figure 2:**
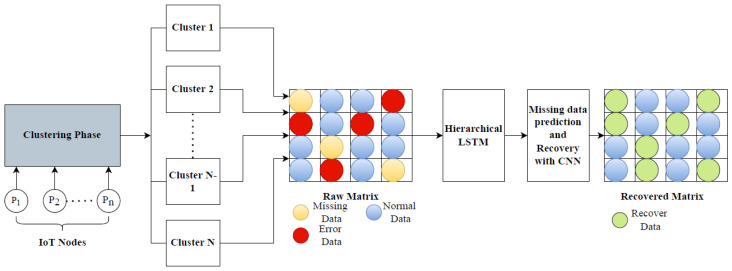
Functional block diagram of proposed approach.

The correlation coefficient gives the statistical relationship between two variables. To enhance the accuracy of correlation coefficient analysis, here spearman correlation coefficient is utilized. Table 3 shows the correlation among the multi nodes, node 9 is considered as centroid for node 2 and node 14. According to the analysis, the correlation between temperature and pressure shows a strong correlation and it is calculated with the following Equation (7)

(7) $$\rho =1-(\left(6\Sigma d_{t}^{2}\right)/\left(n\left(n^{2}-1\right)\right)$$

where ρ is the spearman correlation coefficient, $d_{i}$ is the difference between two ranks of corresponding neighbor nodes, and *n* is the number of observations. Figure 2 shows an illustration of the CLP and DR phases of the proposed method. IoT nodes in the network are grouped into clusters, depending on the spatial correlation between them. Based on the similar sensing range IoT nodes will be clustered together. The raw data from all clusters is a combination of three types of data: erroneous data, normal data, and missing data. A raw matrix is generated which is input to the DR phase, here the ST-HLSTM algorithm is utilized for missing data prediction and recovery. CLP and DR phases are discussed as follows

### 3.4. Clustering Phase

Every IoT node has its data, along with its neighbor nodes, sensing range, and location coordinates. Initially, data are utilized for clustering the nodes which are spatially correlated within the entire network. Algorithm 1 has been implemented for the spatially correlated nodes. The clustering algorithm will be applied based on the type of application and end node mobility. In static networks, single-time clustering is sufficient, but in the case of dynamic networks, clustering should be carried out periodically.

For example, an IoT network consists of a ‘K’ number of nodes, and each node is represented with ‘$P_{i}$’ having ‘N’ neighbors in the network. The first cluster is composed of randomly selected nodes. The selected nodes cannot be allowed to form other groups in the network. At the same time, if a node ‘$S_{i}$’ is a part of the pre-created cluster, that node will not be able to create its cluster. The clustering process is continued until each node in the network belongs to at least one or more clusters. There is a Common Neighbour Node (*CNN*) in between the clusters which are determined in Equation (8).

(8) $$CNN\left(P_{i},P_{j}\right)=\left|N\left(P_{i}\right)\cap N\left(P_{j}\right)\right|$$

where $N\left(P_{i}\right),N\left(P_{j}\right)$ represents as neighbour nodes set of sensor *P_i_* and *P_j_*.

**Figure 3:**
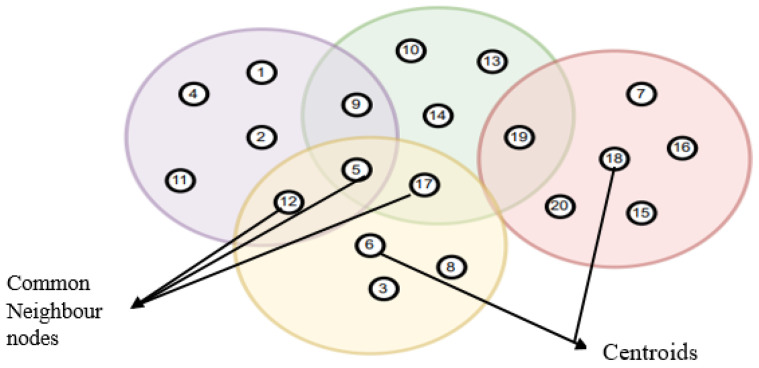
Example for clustering.

As a simple case assume that there are 20 nodes in the network which are randomly deployed. The clustering of nodes has been implemented based on their spatial correlation. The IoT Network nodes 2, 14, 18, and 6 are randomly selected to form clusters by the algorithm. Figure 3 shows that nodes 2, 14, 18, and 6 are reflected as centroids. Nodes 9, 5, 12, 17, and 19 are the common neighbor nodes, and also seen that each cluster has spatially correlated so that node’s data can be utilized interchangeably to retrieve the missing data.
**Algorithm 1** Clustering**INPUT** Total no. of nodes in the IoT network $P=P_{1},P_{2},---,P_{N}$**OUTPUT** List of clusters**Clustering Initialization**1:Set of Clusters list $\left(G_{L}\right)=\left[\right]$2:**for** each node in Network N **do**3:    Select random node $P_{i}$4:    **if** $P_{i}$ already clustered ==1 **then**5:        Return6:    **else**7:        $CL_{d}.append\left(P_{i}\right)$8:        **for** each node $P_{j}\in P\&P_{j}$*isP_i_neighbourdo* **do**9:           $CL_{d}.append\left(P_{j}\right)$10:      **end for**11:    **end if**12:**end for**

### 3.5. Data Recovery Phase

In this phase, the Algorithm 2 is implemented for the prediction and recovery of missing data. By creating the clusters with the help of the CL phase the cluster list has been generated and it will be the input to the DR phase. Initially, the DR algorithm receives cluster lists from the CL phase and converts the list into matrix aXb. Where ‘*a*’ represents the spatially correlated nodes (column) and ‘*b*’ is the past events of each node $P_{i}$ (rows) in the network.

Let us assume that each cluster contains ‘*a*’ number of nodes with ‘*b*’ number of readings. The readings from each node ‘*i*’ at a time ‘*j*’ have been recorded, where *i* = 1, 2, 3, 4 ⋯, *a* and *j* = 1, 2, 3, 4, ⋯, *b*. The readings from each node are required to attain the cluster head appropriately. If any missing or erroneous data are received, then the DR phase will initiate to recover them as follows

Step 1: Initially the readings from each cluster are arranged in the form of a matrix.

$$M_{i}=\left[\begin{array}{ccccccc} R_{11} & R_{12} & R_{13} & \cdot & \cdot & \cdot & R_{1b}\\ R_{21} & R_{22} & R_{23} & \cdot & \cdot & \cdot & R_{2b}\\ \cdot & \cdot & \cdot & \cdot & \cdot & \cdot & \cdot \\ R_{a1} & R_{a2} & R_{a3} & \cdot & \cdot & \cdot & R_{ab} \end{array}\right]$$

where $R_{ij}$ is the readings of each node which are shown in the matrix as rows and columns.

Step 2: Then the missing readings of particular nodes are extracted from the temporally correlated readings of the CNN and represented the relation in Equation (2).

(9) $$Temporalcorrelation\left(t\right)=\left[R_{i1}R_{i2}R_{i3}\cdot \cdot \cdot R_{ib}\right]$$

Equation (3) represents the relationship for entirely spatial correlated nodes readings as

(10) $$Spatialcorrelation\left(l\right)=\left[1/b\sum_{j=1,i=j}^{a}R_{ji}1/b\sum_{j=2,i=j}^{a}R_{ji}\cdot \cdot \cdot 1/b\sum_{j=w,i=j}^{a}R_{ab}\right]$$

Step 3: Matrices *t* and *l* is mean is calculated using the following Equation (4) and it is input as for hierarchical LSTM model.

(11) $$\begin{array}{cc} Mean(M) & =[\sum_{j=1}^{b}\left[1/2\left(t\left(R_{i1}\right)\right)+l\left(R_{i1}\right)\right]\sum_{j=1}^{b}\left[1/2\left(t\left(R_{i2}\right)\right)+l\left(R_{i2}\right)\right]+\\ \cdots & +\sum_{j=1}^{b}\left[1/2\left(t\left(R_{ib}\right)\right)+l\left(R_{ib}\right)\right]] \end{array}$$

## 4. Simulation Parameters and Model Validation Indicators

The data reliability of the proposed method is analyzed through mean absolute error (MAE) and mean squared error (MSE) and compared with other recovery methods such as BI-LSTM, MF-EALS, and DRTSMC. A real-time data set is gathered from a hydraulic test rig. The system periodically repeats regular load cycles (duration 60 s) and calculates process values such as temperatures, pressures, and volume flows while the state of four hydraulic components: accumulator, cooler, pump, and valve is continuously varied. For simulation, one lakh samples have been taken from 51 IoT nodes from a hydraulic test rig. In the data set, 80% of the data are considered training data, and 20% is taken as testing data. Here the selected nodes 1, 5, 11, 14, 18, 24, 39, and 48 are taken as neighbor nodes among all the clusters, and then analyzed the performance metrics of the algorithm accordingly. The impact of the proposed algorithm on an IoT network is evaluated through the following performance metrics.

The proposed algorithms are implemented in google co-lab using python programming with the help of deep learning libraries such as scikit learn, seaborn, Keras, and pandas, etc., The models are trained with a batch size of 20 and 250 epochs to trade off between the convergence rate, training speed, and learning rate is started at 0.01. The tanh activation function is used in the proposed algorithm and it is a non-linear activation function that is used to decide whether that particular neuron is required to be activated or not.
**Algorithm 2** Data Recovery**INPUT** Clusters List $C_{Ld}$**OUTPUT** Recovered Data**Create the Matrices**1:**for** each C $\in C_{Ld}$ **do**2:   create a matrix3:   **if** data is missing then initiates Recovery **then**4:   GR = get New Readings()5:       **for** each $R_{in}\in$ C **do**6:          **if** $R_{in}=null$ **then**7:              *t* = calculate the temporal correlation based on Equation (9)8:              *l* = calculate spatial correlation based on Equation (10)9:              *M* = calculate the spatio-temporal correlation based on Equation (11)10:              ST-HLSTM.input(F)11:              DR=ST-HLSTM.predict()12:              Matrix. append (DR)13:              Return14:          **else**15:              Data transmitted successfully16:        **end if**17:     **end for**18:   **end if**19:**end for**

### 4.1. Model Validation Indicators

To quantitatively differentiate the various data recovery algorithms, here evaluating the metrics such as mean absolute deviation (*MAD*), mean squared deviation (*MSD*), and data reliability (*DR*) are defined as follows:

*MAD* is defined as the average performance error of the proposed approach evaluated. It is used to evaluate the error between actual data and predicted data. *MAD* is calculated with the help of Equation (12). *MAD* of ST-HLSTM attain better results as compared with other data recovery approaches. Meanwhile, the *MAD* of DRTSMC is high as compared to the proposed approach.

(12) $$MAD=1/b\sum_{d=1}^{b}|R_{ji}-{\bar{R}}_{ji}|$$

**Figure 4:**
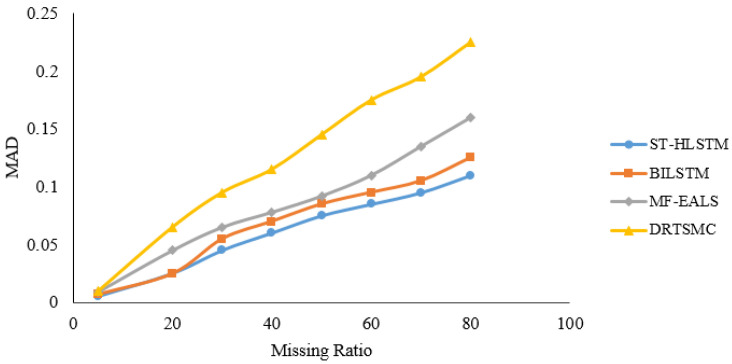
Mean Absolute Deviation for different evaluation methods.

The ST-HLSTM algorithm will consider both spatial and temporal correlation to predict the missing values so, that can able to enhance the data reliability in the network. The temporal correlation is evaluated through the past data of each IoT node. In Figure 4, it is clearly shown that when missing data increases, *MAD* will also increase accordingly, due to the ST-HLSTM prediction can take into consideration both spatial and temporal correlation between the IoT nodes in the network. The temporal correlation is evaluated depending on the past data of each node.

*MSD* is one of the data quality estimators and it is the average square variance between actual and predicted data. *MSD* is calculated by Equation (13).

(13) $$MSD=1/b\sum_{d=1}^{b}{\left(R_{ji}-{\bar{R}}_{ji}\right)}^{2}$$

**Figure 5:**
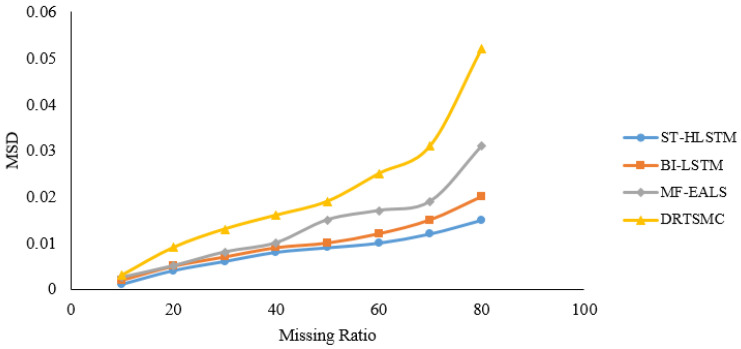
Mean Squared Deviation for various evaluation methods.

The proposed algorithm results in very low *MSD* as compared with the existing algorithms as shown in Figure 5 because the proposed method not only depends on sensor node data correlation but also depends on the correlation between those sensor nodes having similar sensing phenomena. The proposed approach is not only based on node data correlation; it depends on the correlation between the other nodes in the cluster that are having a similar sensing phenomenon. Data reliability is calculated by comparing the real-time sensing values with predicted values with the help of Equation (14).

(14) $$DR=1/b[\left(\sum SD\left(R_{ji}\right)\right)/\left(\sum SD({\bar{R}}_{ji}\right))]$$

**Figure 6:**
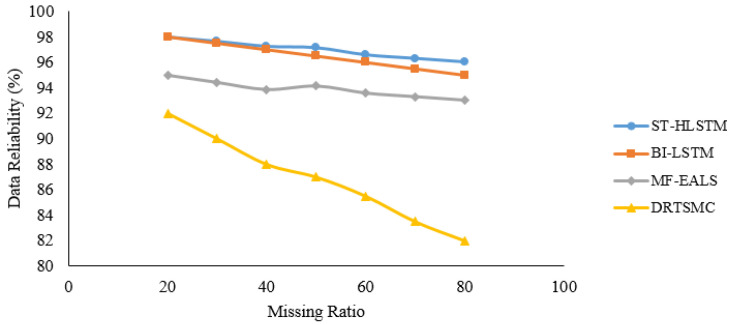
Reliability of Node4.

**Figure 7:**
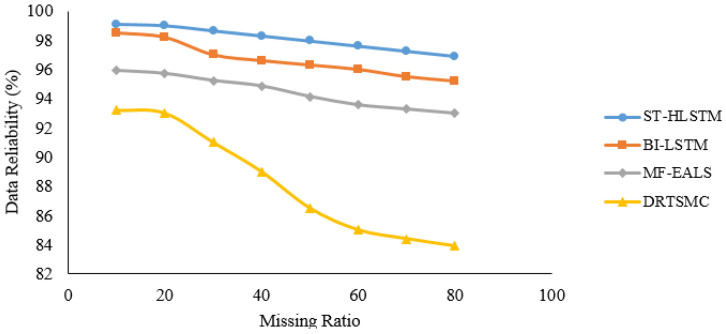
Reliability of Node12.

**Figure 8:**
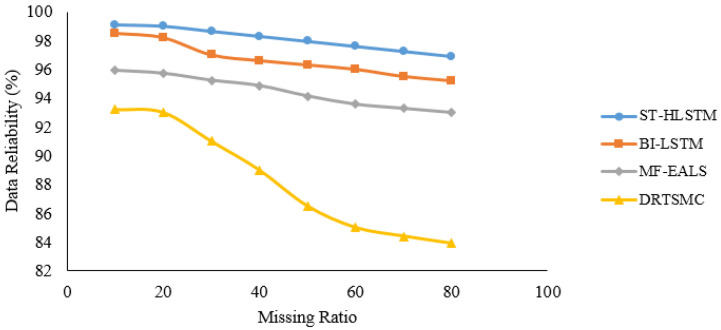
Reliability of Node21.

**Figure 9:**
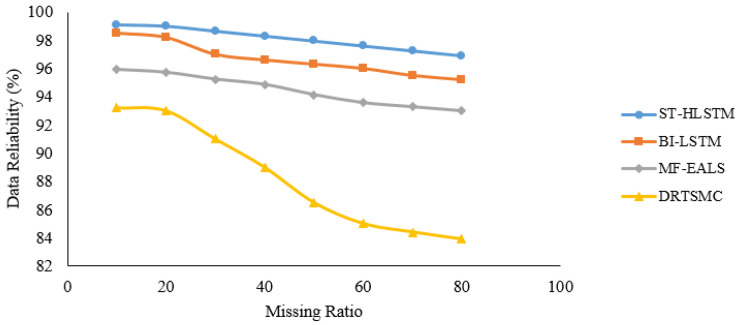
Reliability of Node45.

In Figure 6, Figure 7, Figure 8 and Figure 9, it is evident that ST-HLSTM results in high reliability as compared with the other algorithms. This is because ST-HLSTM is based on spatial and temporal correlation between the IoT nodes and also handles long-term dependencies.

### 4.2. Cost and Complexity Analysis

The data set consists of around 3.5 million data set but for training and testing here randomly considering only 1 Lakh samples for initial testing. The sampling time and interval for the data set are 562 ms and 10 ms. Then min–max normalization is utilized for the randomly selected data set. Later, the data set is divided into three patches whereas the training dataset is 50,000 samples, 20,000 samples are testing and 30,000 samples are blind data sets. To implement various data recovery models Bi-LSTM, MF-EALS, DRSTMC, and the proposed approach ST-HLSTM. The proposed model takes higher training time as compared with existing data recovery models which are mentioned in Table 4. It is one of the limitations of the proposed method.

MF-EALS requires less training and testing time, but this model is applicable for short-term series data, and this is the major limitation, as shown in Table 5. Among all the data recovery approaches, ST-LSTM requires more training and testing time as compared to existing recovery approaches due to its spatial and temporal correlation extraction. The data can be recovered more accurately with the help of the proposed approach.

Table 6 provides the comparison between proposed and existing data recovery approaches in terms of MAD, MSD, and DR. ST-HLSTM approach achieves high data reliability even if the processing data samples are more.

## 5. Conclusions

In this paper, a missing and error data recovery approach is proposed based on HLSTM and ST correlation between the nodes in IoT networks. The proposed recovery approach has two stages: One is clustering, which is used to generate the clusters depending on spatial and temporal correlation between the nodes in the network. In the next stage, ST-HLSTM is implemented for missing data recovery. The results demonstrate the effectiveness of ST-HLSTM in terms of mean absolute deviation, mean squared deviation, and data reliability. The proposed approach is simulated with a real-time hydraulic test rig dataset. This paper considered only local outliers in future work will be focusing on global outliers which occur due to environmental noise which is randomly influencing the collected data. The proposed approach works more effectively and it is cost-effective for implementation as compared to the existing approaches. In the future, we will explore these areas, as well as insights into the performance of the ST-HLSTM algorithm with different data sets in different scenarios.

## Figures and Tables

**Table 1:** Parameters and Definitions.

Parameters	Definitions
t	time step
$h_{t}$	hidden state
$x_{t}$	fusion data at same time step
$o_{t}$	output gate
$f_{t}$	forget gate
$i_{t}$	input gate
$c_{t}$	memory cell
$h_{t-1}$	Previous time step hidden state
$q_{1}$	lower quartile
$q_{2}$	median
$q_{3}$	upper quartile
*d*	original data samples
$d_{min}$	minimum data sample
$d_{max}$	maximum data sample
$d^{l}$	normalized data samples
ρ	spearman correlation coefficient
$d_{i}$	difference between two ranges of corresponding neighbor nodes
*n*	number of observations
K	number of nodes in the network
$p_{i}$	*i*th node
N	neighbors
$s_{i}$	node in the pre-created cluster
$p_{j}$	*j*th node
*P*	total number of nodes in the network
$CL_{d}$	cluster ‘*d*’
*a*	spatially correlated nodes
*b*	past events of each node pi
R	sensor readings of each node
*t*	temporal correlation
*l*	spatial correlation
*M*	mean of the matrices

**Table 3:** Correlation coefficient between nodes.

		Correlation Coefficient	Correlation Coefficient	Correlation Coefficient
S No	Pair of Nodes	(Temperature)	(Pressure)	(Volume)
1	No.9, No. 2	0.9588	0.9674	0.8962
2	No.14, No. 9	0.9825	0.9899	0.9186
3	No. 2, No. 14	0.9647	0.9588	0.8643

**Table 4:** Training time for the multiple data recovery approaches.

S No	Model	Epoch Time (s)	Total Time (h)
1	BI-LSTM	50	5.86
2	MF-EALS	30	4.18
3	DRTSMC	45	5.28
4	ST-HLSTM	55	6.07

**Table 5:** Testing time for data recovery approaches.

S No	Model	Traning Time/Sample (s)
1	BI-LSTM	0.256
2	MF-EALS	0.132
3	DRTSMC	0.172
4	ST-LSTM	0.295

**Table 6:** Comparison of various data recovery approaches.

S.No	Model	Number of Samples Processed	MAD	MSD	DR (%)
1	BI-LSTM	30,000	0.025	0.005	96
2	MF-EALS	15,000	0.045	0.005	95
3	DRTSMC	40,000	0.065	0.009	92
4	ST-LSTM	100,000	0.020	0.004	98.5

## Data Availability

Not applicable.
